# Dendritic cell-based immunotherapy for head and neck squamous cell carcinoma: advances and challenges

**DOI:** 10.3389/fimmu.2025.1573635

**Published:** 2025-05-26

**Authors:** Wenyue Chen, Zhengqiang Li, Jin Tang, Shuguang Liu

**Affiliations:** Department of Oral and Maxillofacial Surgery, Stomatological Hospital, School of Stomatology, Southern Medical University, Guangzhou, Guangdong, China

**Keywords:** head and neck squamous cell carcinoma, dendritic cell, immunotherapy, cancer vaccine, cell therapy, immune checkpoint inhibitors therapy

## Abstract

Head and neck squamous cell carcinoma (HNSCC) is a prevalent cancer with poor response to conventional treatments such as surgery, chemotherapy and radiotherapy. Immune checkpoint inhibitors (ICIs) have revolutionized the treatment of HNSCC, but many patients still exhibit poor responses due to insufficient T cell infiltration and impaired dendritic cell (DC) function within the tumor microenvironment. DCs are crucial for initiating anti-tumor immune responses, but their dysfunction in HNSCC leads to inadequate T cell activation and immune evasion. DC-based immunotherapy offers a promising approach to enhance ICIs therapy efficacy by improving DC function and enhancing T cell-mediated anti-tumor immune response. This review discusses the mechanisms underlying DC dysfunction in HNSCC, recent advances in DC-based immunotherapy, and the potential for combination therapies to overcome resistance to ICIs. Future strategies should focus on optimizing DC vaccines and developing personalized treatments to improve outcomes for HNSCC patients.

## 1 Introduction

Head and neck squamous cell carcinoma (HNSCC) is the sixth most common cancer globally, the overall efficacy of conventional treatments such as surgery, chemotherapy and radiotherapy, is unsatisfactory, with a five-year survival rate of only about 60% for HNSCC patients (1). Immune checkpoint inhibitors (ICIs), especially PD-1/PD-L1 inhibitors, have emerged as a crucial therapeutic modality for HNSCC in recent years. However, a substantial proportion of HNSCC patients exhibit poor response to PD-1/PD-L1 inhibitors (2). The underlying reasons for this phenomenon are multifaceted. First, HNSCC tumors often display insufficient T cell infiltration in the tumor parenchyma compared to normal tissues, a characteristic that has been termed “cold tumors” (3). Second, HNSCC is associated with impaired or inhibited T cell priming (4) and the development of T cell exhaustion (5).

Dendritic cells (DCs) are the key antigen-presenting cells (APCs) for cancer immunosurveillance and the initiation of anti-tumor immune responses (6). Studies have demonstrated that tumor-induced DC dysfunction is one of the major causes of impaired T cell priming, decreased T cell infiltration, and T cell exhaustion (7). Therefore, the lack and dysfunction of tumor-infiltrating DCs are closely associated with poor response to PD-1/PD-L1 inhibitors.

DCs play an integral role for T cell priming in tumors. DCs located in peripheral and internal tissues recognize a variety of antigens. Upon stimulation with tumor antigens, DCs migrate to regional draining lymph nodes (DLNs), where they activate tumor-specific T cells that mediate anti-tumor immune responses (8–10). In HNSCC, however, DC function is severely impaired. Various immunosuppressive cells within the tumor microenvironment (TME), such as tumor cells, myeloid-derived suppressor cells (MDSCs), regulatory T cells (Tregs), and immunosuppressive cytokines, interfere with DC antigen uptake, antigen presentation, maturation, and migration. These interactions prevent DCs from functioning as immune activators and transform them into an immunosuppressive phenotype (11–13), which further leads to T cell dysfunction (9).

Hence, restoration of suppressed DC function is essential to reactivate anti-tumor immunity and increase the response rate of HNSCC to PD-1/PD-L1 inhibitors. It has been shown that DCs in the regional DLNs and tumors of HNSCC are critical for PD-1/PD-L1 inhibitors, and increasing the infiltration of intratumoral DCs significantly improves the response rate to PD-1/PD-L1 inhibitors. DC-based immunotherapy heats up “cold” tumors by increasing intratumoral T cell infiltration, thereby improving the efficacy of PD-1/PD-L1 inhibitors and exerting a synergistic anti-tumor effect. Additionally, it enables the direct killing and clearance of tumor cells by priming and expanding T cells, mediating tumor antigen-specific cytotoxic T lymphocyte (CTL) responses. It is reasonable to conclude that DC-based immunotherapy represents a promising approach for the treatment of HNSCC.

Currently, the application of DC-based immunotherapy in HNSCC is still in its early stages. Initial studies, while obtaining optimistic results, have also led to the emergence of new questions and challenges. For example, it remains unclear how the presence of immunosuppressive DCs and the expression of PD-1/PD-L1 on DCs may interfere with the efficacy of DC vaccines (14, 15). Some technical challenges such as antigen loading, vaccine quality control, and DC preservation also remain to be tackled (16, 17). This article provides an overview of the mechanisms underlying the anti-tumor functions of DCs and their impairment in HNSCC, potential therapeutic targets, recent advances in DC vaccines for HNSCC, and discusses existing challenges and limitations. The aim is to provide new insights for the further clinical translation of potential therapeutic targets of DCs, as well as for the future clinical application of DC-based immunotherapy.

## 2 DCs and their roles in HNSCC

**Figure 1:**
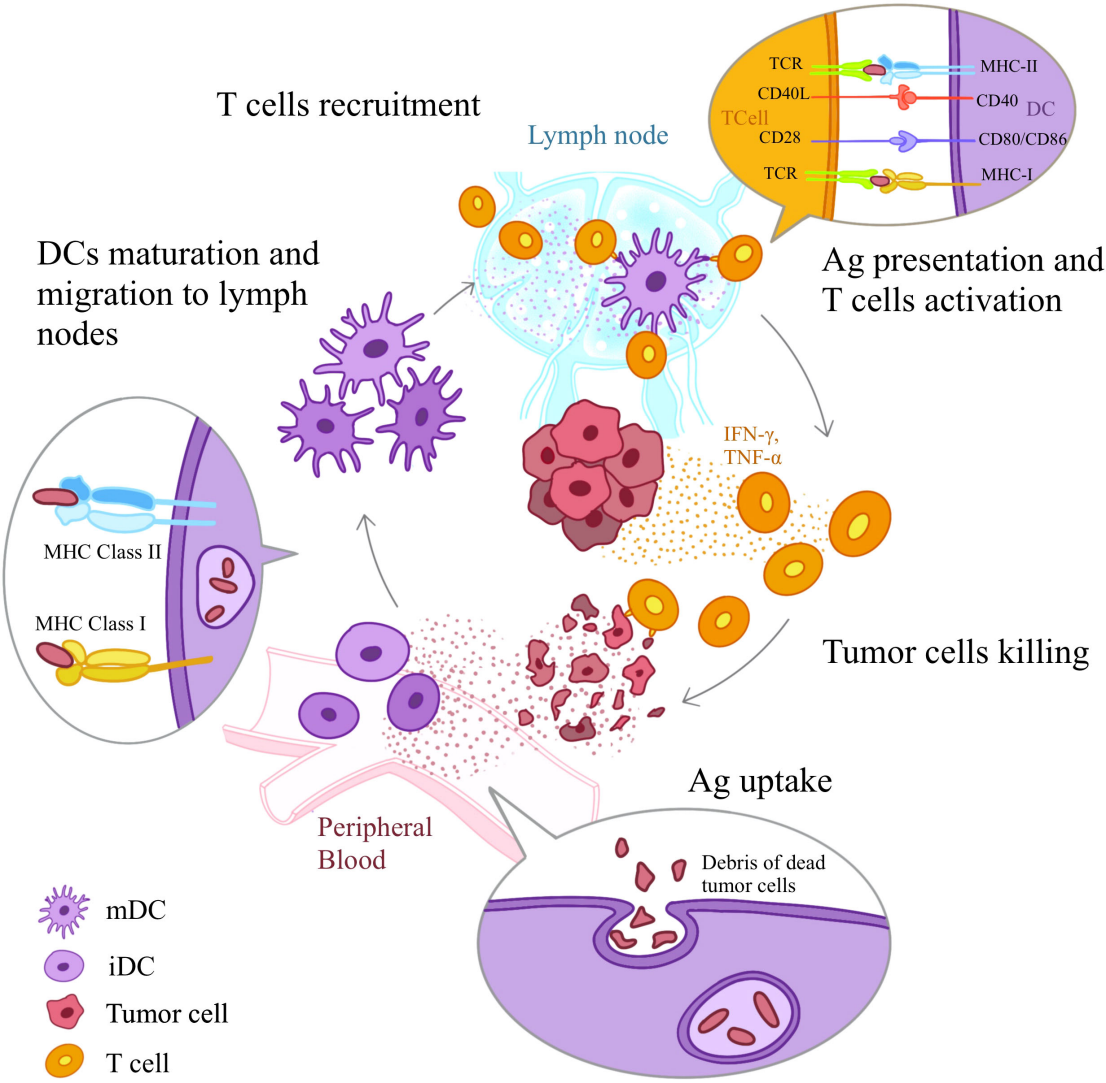
DC-mediated Anti-tumor Immune Response. DCs induce anti-tumor immune responses through a series of steps: Ag uptake, Ag processing, maturation, migration of DCs to the T cell regions of sentinel lymph nodes, Ag presentation to T cells, and T cell activation. First, iDCs detect Ags of dying tumor cells through receptors such as TLRs and PRRs. Next iDCs uptake antigens and initiate the maturation process. With the help of chemokine receptors iDCs migrate spontaneously to secondary lymph nodes. Along the way, they up-regulate the expression of MHC class I and class II molecules and costimulatory molecules (CD80, CD86 and CD40). Once reach lymph nodes, mDCs present Ag-derived peptides to T cells via MHC class I and II molecules and provide the second signals necessary for T cell activation, initiating the activation and differentiation of T cells. TCR, T cell receptor; mDC, mature DC; iDC, immature DC.

In HNSCC, multiple subtypes of DCs exhibit their distinct and unique roles. The most well-studied subtypes are conventional DCs (cDCs) and plasmacytoid DCs (pDCs). cDCs are efficient at antigen presentation, while pDCs are adept at producing type-I interferons (IFNs). In addition to these two subtypes, mature dendritic cells enriched in immunoregulatory molecules (mregDCs), a newly discovered subtype, have been found to be closely related to disease prognosis of HNSCC. DCs in HNSCC are pivotal for initiating anti-tumor immune responses by capturing and processing tumor antigens, migrating to regional DLNs, and presenting antigens to T cells (**Figure 1**) (18–20). However, the immunosuppressive TME often hampers their functions, which further contributes to T cells inhibition. In the context of ICIs therapy, DCs can enhance treatment efficacy by improving antigen presentation and T cell activation, but their function must be restored or optimized to overcome the immunosuppressive barriers posed by the tumor.

### 2.1 DC function and dysfunction in HNSCC

#### 2.1.1 cDC1

The cDC subtype consist of two groups: cDC1 and cDC2. cDC1s exist in various tissues, including blood, lymph nodes, spleen, skin, lung, ileum, Peyer’s patches, intestine, liver, and tonsils. Human cDC1s express multiple TLRs, including TLR1, TLR3, TLR6, and TLR8 (21). However, there is a lack of expression of TLR4 and TLR9 on cDC1s in HNSCC, while they are expressed in mice (22).

The most notable ability of cDC1s is antigen cross-presentation. They uptake antigens from necrotic tumor cells, transport them to draining lymph nodes, and cross-present soluble and particulate antigens via MHC class I molecules (MHC-I) to cancer-specific CD8+ T cells, stimulating tumor-specific CTL responses (23). This unique function relies on the restricted expression of C-type lectin domain family 9A (Clec9a, also known as DNGR-1) (24).

The dysfunction of cDC1 in HNSCC is a key factor contributing to resistance to anti-PD-1 (aPD-1) therapy. The low expression of CD86 and CD40 on cDC1s, along with impaired DC migratory function in aPD-1-resistant HNSCC, contributes to T cell priming inhibition and reduced infiltration of CD8+ T cells in the DLNs. Targeting cDC1 with tumor antigens upregulates CD86 and CD40 expression and promotes their CCR5-mediated migration into DNLs. This leads to CD8+ T cell priming and increased intratumoral infiltration, thereby converting “cold” tumors into “hot” tumors and reversing resistance to aPD-1 (25).

#### 2.1.2 cDC2

cDC2 primarily presents antigens to naïve CD4+ T cells, priming the differentiation of T helper 2 (Th2) cells and Th17 cells via MHC-II presentation (26, 27), and initiates type 3 immune responses by activating ILC3 and Th17 cells.

cDC2 mediates the immune response against human papillomavirus (HPV)+HNSCC. cDC2s can migrate with loaded antigens to the T-B border in the lymph node (28), where they present antigens to CD4+ T cells and induce differentiation into T follicular helper cells (Tfh) (29). Tfh are a specialized subpopulation of CD4+ T cells that help B cells produce antibodies against external pathogens (30). Subsequently, Tfh further activate B lymphocytes to generate an immune response via the CXCL13/CXCR5 axis (31). Notably, only cDC2 can migrate to the T-B border (28), suggesting their integral role in fighting HPV+HNSCC.

In addition, in HNSCC, lactate secreted due to PKM2 upregulation inhibits the NF-κB pathway, thereby promoting Galectin-9-mediated immunosuppression (32). This may contribute to the impaired migration of cDC2 in HPV+HNSCC (33). Moreover, it has now been confirmed that Galectin-9 interacts with PD-1 and TIM-3 to regulate T cell death (34). These findings suggest that Galectin-9 could be a potential target for improving cDC2 migration in HNSCC.

#### 2.1.3 pDC

pDCs, the major producer of IFN-I in HNSCC, detect antigenic RNA and DNA through TLR7 and TLR9, respectively (35–38), and signal through TLR7/9-MyD88-NF-kB pathway (39), leading to pDC maturation, and the secretion of IFN-I (40). As a key component, pDC-derived IFN-I has a broad impact on immune regulation. It enables pDCs to initiate adaptive CTL priming responses, contributes to long-term T cell survival and Th cell generation (41), which are essential to the antiviral immune response.

However, pDCs demonstrate an immunosuppressive function in HNSCC, and high pDCs infiltrations are associated with poor prognosis (42). The immunosuppressive microenvironment in HNSCC inhibits pDC maturation and impairs their capacity to secrete IFN-I. This prevents the proliferation and inhibits the functions of CD4+ Th cells and CD8+ CTLs, while facilitating the infiltration of Tregs (43). In turn, the increasing intratumoral infiltration of Tregs further contributes to immunosuppression, worsening the dysfunction of pDCs. One study indicated that depleting pDCs in HNSCC leads to a decrease in infiltrated Tregs and MDSCs, and enhances tumor control in mouse models (42), suggesting targeting tumor-infiltrated pDCs could be a potential approach to improve HNSCC prognosis.

#### 2.1.4 mregDC

Recently, a new subpopulation of DCs in the TME of HNSCC was identified: myeloid DCs enriched in regulatory molecules, also known as mregDCs, which express IL23A and IL12B. This type of DC controls Tregs and IL-17-secreting T cells in the TME. The Study found that high expressions of IL23A, IL17A, and IL17F are associated with a favorable prognosis in HNSCC. These finding suggested that the expression of IL-23A and IL-12B of mregDCs may contribute to the induction of IL-17 in Th17 and CD8+ T cells (44).

Intriguingly, despite maintaining a mature phenotype, mregDCs are incapable of initiating a sufficient immune response within TME. This phenomenon is associated with the Treg-mregDC crosstalk. The TME is characterized by an abundance of prostaglandin E2 (PGE2), which has been demonstrated to enhance the production of CCL17 and CCL22 by mregDCs via PGE2-EP2/4 signaling (45). Both CCL22 and CCL17 serve as potent chemo-attractants for Tregs and are responsible for CCR4-dependent recruitment of Tregs into the tumor niche (46). Consequently, mregDCs facilitate the accumulation of Tregs around lymphatic vessels in the peripheral tumor stroma, establishing a Treg-mregDC-lymphatic niche (47). Within this peri-lymphatic niche, mregDCs promote Tregs activation as well as the upregulation of their inhibitory molecules and co-stimulatory molecules. In turn, Tregs primarily target mregDCs and inhibits mregDC migration to the DLNs (47). This inhibition effectively restricts the presentation of tumor antigens by mregDCs, ultimately impeding the initiation of anti-tumor immune responses.

### 2.2 Factors contributing to DC dysfunction in HNSCC

TME is a dynamic network system composed of tumor cells, immune cells, fibroblast, endothelial cells, extracellular matrix, cytokines, chemokines and receptors (48). Tumors attract and reprogram DCs to give them immunosuppressive and pro-angiogenic functions (49–51). Additionally, the interactions between DCs, T cells, and MDSCs contribute to the formation of an immunosuppressive environment.

#### 2.2.1 MDSCs hamper DC maturation

MDSCs are a significant regulatory cell population originating from the bone marrow. They exhibit an immature phenotype and exert immunosuppressive effects in the TME (52). Their generation is due to the pathologically activated state of immature myeloid cells and the tumor-induced blockade of their differentiation into DCs or other mature myeloid cells. This blockade is one of the major mechanisms that promote cancer progression (53), and it occurs under cancer conditions and is influenced by tumor-derived factors such as VEGF, IL-6, and IL-1β (54).

Accumulation of MDSCs in TME associates with the inhibition of DC differentiation, which is one of the major immunological abnormalities in cancer and leads to suppression of anti-tumor immune responses. MDSCs infiltration in HNSCC is typically associated with advanced clinical stage and pathological grade, indicating poor overall survival (55). MDSCs are recruited into TME mainly via CCL2/CCR2 axis, CXCLs-CXCR1/2 axis, and CCR5/CCR5 ligand axis (53). In addition, abnormal DC differentiation, resulting from excessive activation of the JAK-STAT3 pathway, also contributes to increased accumulation of MDSCs (56).

What is particularly intriguing about these cells is their complex relationship with DCs. On one hand, MDSCs negatively influence the maturation of DCs through several mechanisms. These include reducing antigen uptake by DCs, blocking of the migration of both iDCs and mDCs, inhibiting of the capacity of DCs to induce IFN-γ-producing T cells, and altering the cytokine production of DCs toward an anti-inflammatory phenotype (12). The production of the pro-inflammatory cytokine IL-23 by DCs and its downstream induction of Th17 cells may contribute to the effects of MDSCs on DCs (57). However, these mechanisms have not been fully investigated.

On the other hand, under certain conditions, MDSCs can be induced and transformed into mature myeloid cells, including mDCs, thereby breaking the suppression (58). The IFN-α produced by pDCs upon the stimulation of cytosine-phosphorothioate-guanine (CpG) plays a major role in promoting the maturation of MDSCs. Since CpG is an agonist for TLR9, it can be applied as an adjuvant for DC vaccine (59). A study of breast cancer in a murine mammary carcinoma model showed that increased extracellular release of Heat shock protein 70 (Hsp70) induces MDSCs to differentiate into mature DCs, alleviating MDSC-mediated immune suppression and ultimately activating a potent anti-tumor immune response (60). Since Hsp70 is also expressed on HNSCC cell membranes (61), it is reasonable to speculate that the same mechanism is also existing in HNSCC. These findings provide a molecular basis for inducing the differentiation of MDSCs into mDCs, potentially serving as a novel strategy to enhance the effectiveness of DC-based immunotherapy for HNSCC.

#### 2.2.2 Nature killer cells contribute to DC maturation, but their number decreases in HNSCC

NK cells are significantly decreased in HNSCC (62), which may contribute to the abnormal DC maturation and hamper the clearance of iDCs. NK cells promote DC maturation and enhance their ability to stimulate naïve CD4+ T cells through cell-cell contact (63) and the secretion of soluble factors such as TNF-α and IFN-γ. Intriguingly, NK cells also induce the lysis of iDCs (58). However, the underlying mechanism remains poorly understood, and it is not yet clear under what circumstances iDCs would further mature or be killed by NK cells.

### 2.3 DC dysfunction contributes to T cell exhaustion and “cooling” the tumor

#### 2.3.1 Immunosuppressive DCs inhibit T cell function and induce Tregs

The antigen cross-presentation function of DCs is of paramount importance for the generation of anti-tumor T cells, as previously highlighted. However, a growing body of evidence suggests that, in addition to their beneficial effects, DCs can sometimes exert an opposing influence within the TME (64, 65). The modification and inhibition of tumor-infiltrating DCs by the TME compromise their ability to initiate robust anti-tumor immune responses and may even facilitate tumor progression. Consequently, tumor-infiltrating DCs induce T cell tolerance rather than immunity via tumor antigen cross-presentation in tumor-bearing hosts (66).

LAMP3+cDCs represent a mDC subset with an “active” phenotype and a higher migratory propensity, identified in the TME of various tumor types by Cheng et al. In nasopharyngeal carcinoma (NPC) the infiltration of LAMP3+cDCs in tumors is significantly increased than in normal tissues (67). LAMP3+cDCs interact with T cells through the PD-1/PD-L1 (68). CMTM6, a protein that can reduce the half-life of PD-L1, is highly expressed in LAMP3+ cDCs (67). Moreover, LAMP3+ DCs also express PDCD1LG2, CD274, NECTIN2, CD80, and CD86, which are immune checkpoints targeting CTLA-4, TIGIT, and PDCD1 thus inhibiting CD8+T cell activation (69). Additionally, cDC1-derived LAMP3+cDCs promote the differentiation of Tregs by expressing higher levels of BTLA, while cDC2-derived LAMP3+cDCs express chemokine CCL17 to recruit Tregs into the TME (68). Collectively, through these mechanisms, LAMP3+cDCs contribute to T cell exhaustion and mediate immunosuppression within the TME.

#### 2.3.2 DC maturation dysfunction leads to T Cell priming inhibition

The activation of T cells during an immune response requires two signals. The first signal comes from the T cell receptor (TCR) recognizing MHC/antigen peptide complexes, the second signal is provided by co-stimulatory molecules on DCs. Without the second signal, T cells cannot be effectively activated and may instead undergo apoptosis, leading to immunosuppression. Immature DCs (iDCs) are unable to provide the essential signals for T cell priming due to the low expressions of MHC I and II molecules and co-stimulatory molecules (70). Only during the maturation, do DCs upregulate the expressions of these molecules. Thus, mDCs are essential for T cell priming (71).

In peripheral circulation, antigens are captured and internalized by iDCs and activate maturation in normal conditions (72). However, in the immunosuppressed environment of HNSCC, important maturation-related receptors are inhibited, such as TLRs, causing maturation dysfunction. Consequently, these dysfunction iDCs or semi-mature DCs mediate immunosuppression or immune tolerance (73) through several mechanisms:

First, dysfunction iDCs inhibit T Cell priming and regulate T cell anergy and apoptosis by presenting antigens to T cells with the absence of co-stimulatory signals (74), interacting with T cells via PD-1/PD-L1 axis, and secreting of TGF-b1 (75).

Second dysfunction iDCs promote the proliferation of T cells toward phenotypes that contribute to immunosuppression. mDCs are capable of secreting cytokines (e.g., IL-12) that drive T cell differentiation into effector cells (e.g., Th1 and CTL). In contrast, DC maturation dysfunction results in decreased secretion of these cytokines, thereby altering the direction of T cell differentiation and leading to the generation of Tregs (74).

Third, iDCs secrete fewer immunostimulatory cytokines compared to mDCs (76), while releasing higher levels of immunomodulatory cytokines such as IL-10 and TGF-b and enzymes like indoleamine 2,3-dioxygenase (IDO) and heme-oxygenase-1 (75). As a result, iDCs creates an immunosuppressive environment that prevents the immune system from detecting and eliminating tumor cells, thereby facilitating tumor immune evasion (77). Meanwhile, the impaired functionality of T cells also contributes to the poor response of HNSCC to ICIs therapy.

#### 2.3.3 DC migratory dysfunction leads to a decrease in antigen-specific CD8+T cells

After antigen uptake, DCs migrate spontaneously to DLNs under the mediation of chemokines (78). mDCs upregulate chemokine receptors CCR5, CCR7, CXCR4, and chemokine co-receptor CD38, which is essential for the proper functioning of chemokine receptors (79, 80). In DLNs, mDCs present antigen to T cells, thereby activating an immune response.

Current findings have confirmed the importance of cDC1s in the DNLs in mediating an effective antigen-specific CD8+T cells anti-tumor response, and in establishing a favorable response to ICIs therapy in HNSCC (81, 82). However, in HNSCC the expression of CCL5 was insufficient, and it is associated with resistance to aPD-1 therapy (25). CCL5 is an important chemokine that mediates the migration of DCs, and its insufficient expression may have impaired the migratory function of DCs, leading to a reduction of functionally normal CDC1s in DNLs. This impairs the antigen trafficking and presentation process, resulting in decreased antigen-specific CD8+ T cell infiltration and contributing to the resistance against aPD-1 therapy.

## 3 Leveraging DC functions for HNSCC treatment

### 3.1 TLR-mediated DC modulation for HNSCC therapy

In the peripheral circulation, antigens are captured and internalized by DCs through phagocytosis, pinocytosis, and receptor-mediated endocytosis (72). TLRs expressed on DCs are a type of pattern-recognition receptor (PRR) that enables DCs to recognize pathogen-associated molecular patterns from pathogens and tumor cells. TLR activation has profound effects on DCs, influencing various aspects such as endocytosis, cytoskeletal rearrangement, migration, antigen processing, and antigen presentation. Surface TLRs (TLR 1, 2, 4, 5, 6, and 11) bind to common structural components of tumor cells to mediate antigen endocytosis, while intracellular TLRs (TLR 3, 7, 8, and 9) detect nucleic acids, inducing both inflammatory responses and IFN-I responses (83, 84). Once triggered by tumor or pathogens (19), TLRs signal through two major pathways, MyD88-dependent pathway and TRIF-dependent pathway (84), leading to the production of pro-inflammatory cytokines and the initiation of immune responses. Given the important roles TLRs play in DC activation, they are considered potential therapeutic target for HNSCC treatment.

#### 3.1.1 TLR2

The functions of TLRs in DCs are intricate. Sometimes, their activation not only mediates the antitumor functions of DCs, but also impairs the normal functions of DCs. Versican is a large chondroitin sulfate proteoglycan, and also serves as a TLR2 ligan. It has been found to be more strongly expressed in the stroma of local metastases and in the earlier stages of disease in pharyngeal squamous cell carcinoma (PSCC) (85). A study demonstrated that versican induces upregulation of IL-6R and IL-10R expression on DCs through TLR2 activation, thereby enhancing the sensitization of DCs to IL-6 and IL-10. In contrast, IL-10 and IL-6 reprogram sensitized DCs to an immunosuppressive phenotype that produces IL-10. Additionally, the inhibition of TLR2 results in alleviation of DC dysfunction (86), which could be a potential strategy to alter DC dysfunction in PSCC.

#### 3.1.2 TLR3

TLR3 is a receptor for double-stranded RNA selectively expressed in cDCs. TLR3 triggers cDC maturation and the production of cytokines, chemokines, chemokine receptors, co-stimulatory molecules, MHC molecules, and a series of signaling molecules during maturation (87), contributing to antigen cross-presentation and the induction of T cell responses. TLR3 can be upregulated by viral infection and exogenous poly(I:C), triggering a strong IFN-I response (88). Therefore, it could potentially be targeted for reversing DC migratory dysfunction.

#### 3.1.3 TLR4

TLR4 is well known for recognizing lipopolysaccharides (LPS) on the outer membrane of bacteria (89). However, it also demonstrates anti-tumor functions. Evidence has shown that DCs require signaling through TLR4 and its adaptor MyD88 for processing and cross-presentation of antigens from dying tumor cells during chemotherapy or radiotherapy (90). Therefore, it is indeed worth discussing whether targeting TLR4 might enhance the efficacy of chemotherapy or radiotherapy.

#### 3.1.4 TLR9

TLR9 is a known receptor for detecting bacterial and viral unmethylated CpG-DNA (36), and mediates DC maturation in a MyD88-depending manner (18). Similar to TLR4, a recent study on HPV-associated tumors bearing mice showed that targeting TLR9 is also capable of initiating DC-mediated anti-tumor immune response following chemotherapy and radiation therapy (91, 92). TLR9 senses tumor DNA that released following chemotherapy and radiation therapy, and facilitates DC accumulation, antigen uptake, and DC maturation in the TME. It also promotes the migration of DCs into the DLNs to generate tumor-specific CTLs. However, these studies were conducted in mice, and additional validation is needed for targeting TLR9 in human HNSCC, as TLR9 expression is lacking in human in this context (93). In addition, it is unclear whether this mechanism exists only in HNSCC following radiotherapy and chemotherapy.

### 3.2 Non-TLR DC activation pathways in HNSCC therapy

#### 3.2.1 Clec9a

Clec9a is a member of the C-type lectin domain family and is restrictedly expressed on cDC1 (94). Clec9a on the cDC1 surface can bind to F-actin exposed by dying tumor cells (95). Subsequently, the surface Clec9a is internalized and diverts phagocytosed dead cell cargo to a non-degradative recycling endosome compartment. Clec9a activates Syk and NADPH oxidase causing phagosomal rupture, which leads to the release of phagosomal contents into the cytosol and subsequent presentation via MHC-I. This process facilitates cross-presentation of dead cell-associated antigens to CD8+ T cells and induces an efficient CTLs response (96). Clec9a may serve as a potential therapeutic target that enhances the efficacy of aPD-1 therapy by improving the function of infiltrating cDC1 cells in HNSCC.

#### 3.2.2 RIG-I-like receptors

HPV+ HNSCC is associated with the long-term viral infection, particularly high-risk HPV16 (97). Other high-risk subtypes, such as HPV18 and 33, are most strongly associated with oropharyngeal cancer, while HPV52 is associated with oral cancer (98).

RIG-I-like receptors (RLRs) are the receptors of viral RNA, and are key mediators of immune responses in HPV-associated carcinomas. Both cDCs and pDCs express cytosolic RLRs, including RIG-I, MDA5, and LGP2. Both RIG-I and MDA5 are capable of inducing the production of IFNs and pro-inflammatory cytokines that mediate innate immune responses against virus (99). In HPV infections, the vial oncoproteins E6, especially those from high-risk HPV16, partially impair innate immune signaling pathway RIG-I/MAVS/TBK1, leading to an unresponsive cellular state that is conducive to viral persistence and tumorigenesis (100). RIG-I agonist M8 can be reactivate this pathway and triggers the production of large amounts of type I/III IFN, thereby enhancing chemotherapy-mediated cell killing (101). Although LGP2 does not induce the production of IFNs directly, it promotes the survival and cytokine production of activated CD8+ T cells in virus infection (102).

Interestingly, RLRs also exhibit anti-tumor effects in non-virus associated cancer, which has been confirmed by numerous studies. Selective activation of RIG-I has been demonstrated to enhance the infiltration of CD8+ and CD4+ T cells with antitumor function in breast cancer, and has the potential improve the response to immune checkpoint inhibitors and other immunotherapeutic modalities (103). Moreover, another study on breast cancer revealed that MDA5-mediated type-I IFN production is essential for the cross-priming capability of DCs after radiotherapy; its absence impairs ionizing radiation-induced IFN-γ production by CD8+ T cells (104). Although no studies have yet examined whether targeting RLRs have a therapeutic effect on HNSCC, given that Tregs in HPV+/- HNSCC express IFN-α signaling and general IFN response genes early during differentiation (105), it is worth discussing whether therapies targeting RLRs can boost DC cross-priming function, reduce early-activated subpopulations of Tregs, and enhance the effectiveness of radiotherapy in HNSCC patients.

## 4 DC-based Immunotherapy for HNSCC

**Figure 2:**
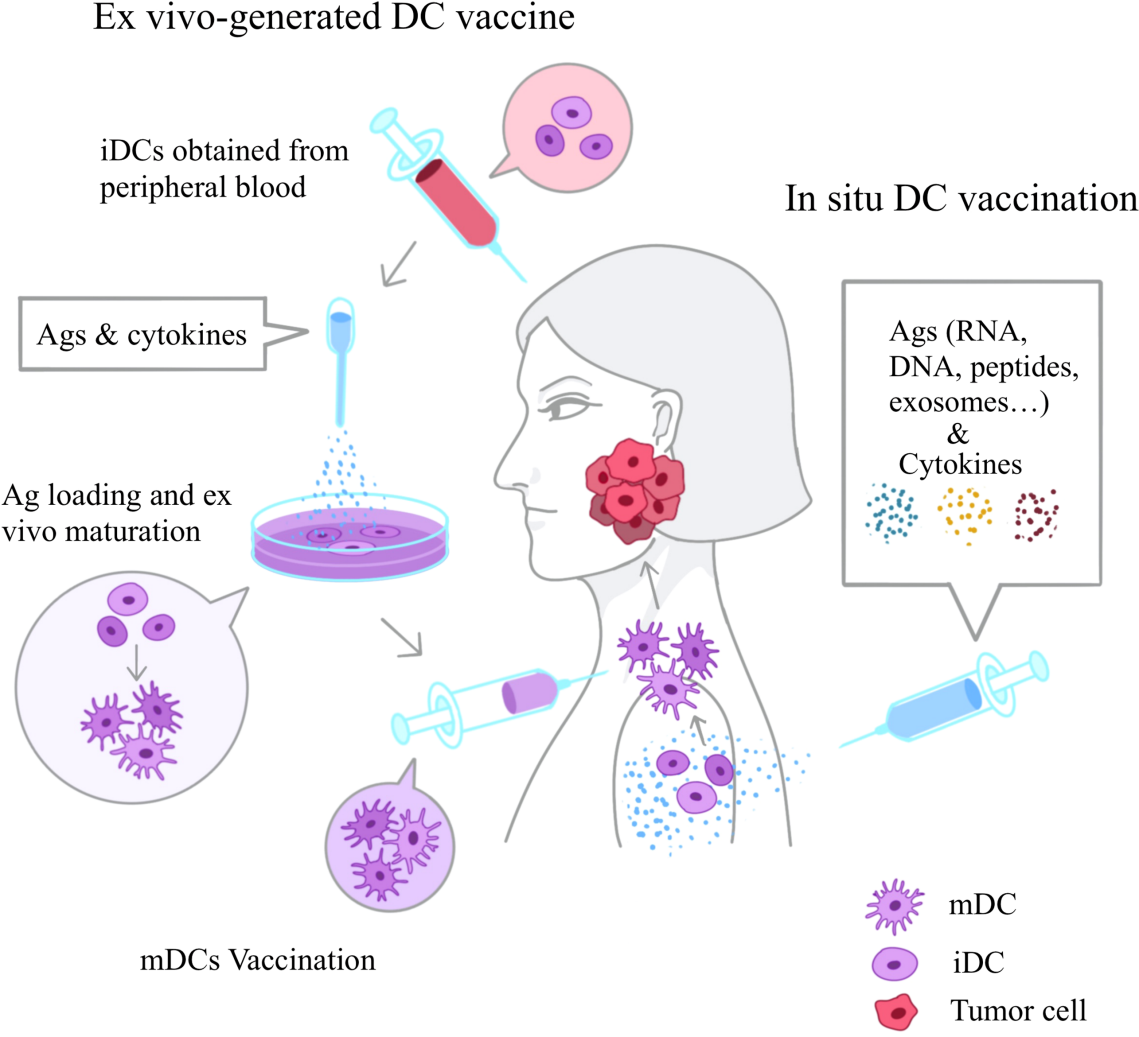
Two Basic Types of DC Vaccines. DC vaccines can be roughly divided into two types, *in situ* DC vaccination and ex vivo generated DC vaccine. *In situ* DC vaccination can directly target the activation of DCs present in the lesion or in lymph nodes at specific sites by injecting drugs, RNA, exosomes or nanoparticle-loaded therapeutic agents. And recently exosomes, whether are cell-originated or engineered, are also used as *in situ* vaccines. Ex vivo vaccination is based on PBMCs isolated from peripheral blood. These personalized PBMCs are induced to differentiate into iDCs ex vivo with exogenous cytokines such as IL-4, GM-CSF and CD40L. And then they are loaded with Ags to become fully mature DCs and reinjected back to the patient. Ag, antigen; iDC, immature DC; mDC, mature DC.

In cancer, the efficacy of cytotoxic T cell immunity is significantly constrained if there is an insufficient number of DCs infiltrating in the tumor and the regional DLNs. DC-based immunotherapy addresses this issue through a variety of strategies, including increasing the number of infiltrating DCs, inducing DC activation, or reducing the presence of inhibitory immunosuppressive DCs. Currently, a diverse array of DC-based immunotherapy approaches exists. They vary from the selection of antigens for vaccines, the methods of antigen loading, the choice of adjuvants, to the routes of administration. However, in general terms, DC vaccines can be classified into two main categories: *in situ* DC vaccination and ex vivo-generated DC vaccines (**Figure 2**) (106, 107).

### 4.1 Tow major approaches for DC vaccines preparation

#### 4.1.1 *In Situ* DC vaccination

*In situ* DC vaccination is designed to directly target the activation of DCs present in the lesion or in lymph nodes at specific sites, as well as their migration to the draining lymph nodes. There, they present the antigens to immune cells (108). To date, several agents have been developed for targeting DCs *in situ*, including the use of cytokines, RNA, exosomes, and nanoparticle-loaded therapeutic agents.

#### 4.1.2 *Ex Vivo* DC vaccination

In contrast to *in situ* vaccination, which is susceptible to interference from *in vivo* factors such as the immunosuppressive environment of the TME, ex vivo-generated DC vaccines employ a distinct strategy. This approach is based on the isolation of DCs from peripheral blood mononuclear cells (PBMCs). These personalized PBMCs are treated with exogenous cytokines like IL-4, granulocyte-macrophage colony-stimulating factor (GM-CSF), and CD40L to be induced into mDCs, then loaded with antigens/adjuvants and reinjected into the patient (109).

The first generation of ex vivo-generated DC vaccines used monocytes cultured with GM-CSF and IL-4 to generate monocyte-derived DCs (moDCs), which had a poor outcome, with tumor regression rate of 3.3% (110). In the second generation, maturation cocktails were introduced in the vaccine, as a result the median overall survival of this generation increased by 20% (110). Third generation DC therapy focuses on specific DC subtypes such as pDCs and cDCs. They are selected from naturally occurring DCs for their different functions in immune activation and antigen presentation (110). This more refined vaccine development strategy helps to avoid interference with therapeutic efficacy caused by immunosuppressive DCs, resulting in more controlled and predictable efficacy.

#### 4.1.3 Strengths and limitations of the two approaches

*In situ* vaccination activates DCs directly in the patient through therapeutic agents. However, the influence of the internal environment of HNSCC patients on DCs is unpredictable. The tumor creates an immunosuppressive-dominant environment, characterized by the presence of a wide range of immunosuppressive cells and cytokines. Additionally, the extremely complex crosstalk between DCs and other cells in the TME makes it difficult to effectively rule out factors that may interfere with this therapy.

In contrast, ex vivo vaccination induces DCs in a controlled *in vitro* environment, which allows for the creation of conditions favorable to DC maturation and maximizes the exclusion of interference from immunosuppressive factors. Notably, however, DCs induced *in vitro* must eventually be infused back into the patient. Since DCs are living cells, they will still respond to various stimuli, and their phenotype may shift. These changes remain unpredictable and uncontrollable at present.

Both *in situ* and ex vivo vaccination approaches have been studied in the treatment of HNSCC. Although the current amount of clinical data is insufficient to scientifically analyze the efficacy of the two approaches in HNSCC treatment, comparing the external influences on DCs in the two approaches suggests that *in situ* vaccination may face more potential negative interferences in its application.

In terms of safety, neither *in situ* nor ex vivo vaccination has resulted in serious adverse events. However, the preparation of ex vivo DC vaccines requires the collection of peripheral blood from the patient, which might lead to some associated adverse effects. Most patients with advanced HNSCC have complications such as cachexia and anemia, and therefore may not be suitable for this treatment strategy. Insufficient blood collection may also result in the failure of vaccine preparation (111). In addition, compared with ex vivo DC vaccines, *in situ* DC vaccines are easier to prepare, have a shorter production cycle, and have lower production costs, and may therefore be more efficiently deployed in clinical applications.

### 4.2 Current DC vaccines strategies for HNSCC

#### 4.2.1 Single-target DC vaccines strategies

There are multiple therapeutic targets in tumors, and the common strategy for tumor-targeted therapy is to bind them with antibodies and inhibit their effects. However, the efficacy of this single strategy is often limited. DC-based immunotherapy provides a new approach for tumor targeting, which involves mediating the recognition and killing of tumor cells that overexpress specific targets by T cells, thereby enhancing tumor control. This new strategy has been tested in the treatment of HNSCC. The epidermal growth factor receptor (EGFR) is overexpressed in over 80% of HNSCC patients, and its overexpression is associated with a poor prognosis (112). A recombinant glutathione-S-transferase-EGFR fusion protein-pulsed DC vaccine has been developed with the aim of targeting EGFR+ HNSCC. Results showed that the vaccine effectively induced high cytotoxicity of CTL responses against EGFR+ cancer cells. Significant anti-tumor immunity was observed, characterized by increased IFN-γ secretion from T cells, a marked reduction in tumor volume, and prolonged survival (113). Several studies have been conducted on this single-target DC vaccine strategy for HNSCC. For example, a Wilms Tumor 1 (WT1) peptide-pulsed DC vaccine, which targets a common WT1 gene mutation in HNSCC (114), and a p53 peptide-loaded DC vaccine, targeting the p53 gene (115), are already undergoing clinical trials and will be described in detail in the next section.

In addition, DC vaccines possess distinct advantages over traditional targeted therapies. There are certain molecules that are exclusively overexpressed in tumor cells rather than in normal tissues. However, their specific roles in tumor progression and the underlying mechanisms remain unclear. As a result, these molecules are currently utilized solely as prognostic markers, and no drugs are available to target them. In contrast, in DC-based therapy, these molecules can be prepared as antigens for loading onto DCs, in order to induce antigen-specific CTL responses for targeted killing of tumor cells.

#### 4.2.2 Multi-target DC vaccines strategies

HNSCC is a heterogeneous disease, and mutations within it occur frequently, leading to high variability in the expression of its molecules. This variability is highly likely to affect the effectiveness of a DC vaccine targeting a single antigen. Therefore, increasing the number of targets for DC vaccines as much as possible will help to maintain the stability of therapeutic efficacy. This requires that the antigens used for pulsing DCs have an antigenic spectrum that is as broad as possible. Currently, the commonly used antigen sources in the design of this type of DC vaccine for HNSCC are whole tumor cells and cell lysates.

Hybrid DC vaccines is prepared by fusing DCs with tumor cells, which allows for the introduction of antigenic information into DCs. Autologous whole tumor cells encompass the full spectrum of a patient’s tumor antigens, including mutated neoepitopes, eliminating the need for individual antigen identification (116). As early as 2004, a study confirmed the feasibility of producing hybrid cells using DCs and human laryngeal cancer tumor cells via electrofusion (117). Subsequent research by Lee et al. showed that DC-SCC7 cell hybrids vaccine provided significant protection tumor bearing mice; after subcutaneous vaccination, a reduction in tumor size (*P*<0.001) was observed. Moreover, after the introduction of anti-OX40R monoclonal antibodies as an adjuvant, survival rates in pulmonary metastasis and subdermal tumor models were both increased (118). However, the high cost of electrofusion and high technical challenge of electrofusion poses challenges for its large-scale clinical application (119).

Tumor cells can be prepared into lysates, which also allows the loading of a wide variety of antigens to DCs to ensure maturation, and are capable to induce both CD4+ and CD8+ T cell response (120). As one of the most commonly used antigen sources for DC vaccines, tumor lysates offer the advantages of simple preparation and lower cost. However, DCs loaded with tumor lysates must first overcome technical challenges that may hamper vaccine efficacy. These challenges include the instability and inefficient internalization of soluble macromolecules, which are major issues for antigen transportation and cross-presentation to T cells. In the development of a DC vaccine pulsed with FaDu cell and FAT7 cell lysates, a nanoparticle delivery system composed of poly(lactic-co-glycolic acid) (PLGA) copolymers was introduced to address these challenges (121). The increased efficiency of antigen delivery to CD8+ T cells attributed to PLGA nanoparticles has been demonstrated. However, since tumor cell lysates also contain immunomodulatory cytokines from the tumor cells, which cannot be isolated during preparation, a tolerogenic transformation of DCs may be induced (120), thus limits the application of tumor cell lysates.

#### 4.2.3 Universal cancer vaccine strategies

Since HNSCC follows the universal pathological mechanism and shares some common targets with many tumors, we herein review some of the *in situ* DC vaccination approaches that have the potential to be applied in HNSCC.

A *in situ* DC vaccine based on albumin fused with FMS-related tyrosine kinase 3 ligand (Alb-Flt3L) has been proven to improve tumor control and overall survival. Alb-Flt3L is a Flt3L-based immunotherapy used in combination with chemoradiation. Its core function relies on the expansion of cross-presenting DCs and the synergistic effects of chemoradiation on immunotherapy (122). Flt3L is a key cytokine mediates the differentiation of haematopoietic progenitor cells into cDCs and pDCs. Fusing Flt3L to Alb promotes its accumulation in tumor and lymphatic nodes (123). In the presence of the Alb-Flt3L, cDC1, which is crucial for antigen cross-presentation, is expanded in a BATF3-dependent manner (122). At the same time, chemoradiation induces cell death and apoptosis, thereby increasing the release of tumor antigens. This provides greater access to antigens for uptake and cross-presentation by Alb-Flt3L-expanded DCs. Since DCs are not pulsed by specific tumor antigens, therefore, Alb-Flt3L can be widely applied to a wide range of cancer types.

RNA vaccine is universally approach that is applicable to most types of malignancies, since all tumor-associated antigens (TAAs) can be encoded by the corresponding RNA. An RNA lipoplexes (RNA-LPX) vaccine has been developed by loading RNA onto LPX, a lipid carrier. This protects RNA from extracellular ribonucleases and ensures that the coding antigens are efficiently taken up and expressed by cDCs and pDCs following intravenous injection. RNA-LPX vaccine has demonstrated a high efficacy in inducing robust antigen-specific T-cell responses in phase I clinical trial (NCT02410733) (124). Although the trial was conducted on melanoma patients, two of the RNA-LPX vaccines tested encoded MAGE-A3 and NY-ESO-1, respectively, which are also expressed in HNSCC. MAGE-A has been shown to impair p53 function (125), potentially leading to decreased chemosensitivity and radiosensitivity (126). MAGE-A3 expression in HNSCC is associated with decreased overall survival, since in conventional therapy, the immune system appears unable to counteract the aggressiveness of MAGE-positive tumors (127). Therefore, antigen-specific therapy in the form of RNA vaccines may be helpful in improving responses to chemotherapy and radiotherapy in HNSCC. NY-ESO-1 is one of the most immunogenic proteins described in human cancer, capable of inducing simultaneous antibody and CD8+ and CD4+T cell responses. However, its expression rate is too low for clinical application, though it might be a candidate for immunotherapy and poly-vaccination in HNSCC patients (128). In addition, engineered nanoparticles that can be use as RNA carrier to enhance the therapeutic efficacy of RNA vaccine by facilitating targeted delivery and immune modulation (129). Multiple nanoparticles, such as ionizable lipid nanoparticles (LNPs) (103), PLGA nanoparticles (130), have been used as RNA carriers to enhance the therapeutic efficacy of RNA vaccines by facilitating targeted delivery and immune modulation. These nanoparticles have demonstrated highly stable, loadable, and sustained-release features, as well as the ability to target DCs. However, it is worth noting that tumor-derived mRNA allows the transfection of TAAs and co-stimulatory molecules, ensuring antigen presentation in MHC-I without requiring cross-presentation, but it cannot induce a potent CD4+ immune response (120).

Tumor-derived exosomes (TEXs) carry a variety of proteins such as MHC-I and MHC-II, phosphatidylserine, milk fat globulin-E8 (MFGE8), rab7, liposome-associated membrane protein 1 (LAMP1), CD9, CD81, Annexin II, CD54, and CD63 that facilitate exosome-binding and uptake by relative ligands on DCs (131). They also contain a wide spectrum of TAAs and demonstrate immunogenicity for activating DCs (132). When TEXs are loaded onto DCs, they transfer shared tumor antigens and initiate DC maturation, allowing for the specific activation of CTLs both *in vitro* and *in vivo* (133). Meanwhile, there is growing evidence revealed in the studies on other cancers, that mDCs induced by TEXs mediate stronger CD8+ CTL responses and exhibit enhanced anti-tumor effects compared to cancer cell lysates, which are currently the most widely used for pulsing DCs (134–136).

However, HNSCC-derived exosomes can also contain other immunosuppressive molecules from parental tumor cells, such as DNA, mRNA, and microRNAs (137). When they transfer these molecules to DCs, they reprogram DC functions and promote tumor progression (138). Therefore, current studies on HNSCC-derived exosomes have focused on its mediation of tumor immune escape and less attention has been paid to its effect on DC maturation. Notably, most of the exosomes that are currently used as antigens for DCs can be engineered to eliminate their immunosuppressive properties and to acquire characteristics that enhance immune activation performance (139). Hence, HNSCC-derived exosomes can be made a potential antigen source for DC vaccines by combining them with the engineering technics.

#### 4.2.4 Combination therapies for HNSCC

DC-based immunotherapy is an excellent adjunct to ICIs therapy due to its inherent ability to mediate T cell immune responses. The activation of DCs in HNSCC have now been shown in multiple studies to be associated with improving response rate to ICIs therapy (25, 81, 82), and the mechanisms have been discussed in detail in this review. The combination of DC vaccines and ICIs therapies is currently being explored in several clinical studies (**Table 1**). Although there is not enough data available at this point in time, it is a strategy with optimistic prospects, considering the promising outcomes it has obtained in other cancer treatments (140).

**Table 1:** Clinical trials of DC-based immunotherapy for HNSCC registered in ClinicalTrails.gov.

Trail ID	Strategies	Indication	Phase	Status	Antigen	Combinatorial treatment
NCT00377247	Autologous DC vaccination	Carcinoma of Oral Cavity or Oropharynx	I	TERMINATED	autologous tumor DNA	-
NCT00404339	Autologous DC vaccination	HNSCC	I	COMPLETED	wild-type p53 peptides, T-helper peptides	–
NCT00492947	Autologous DC vaccination	HNSCC	I	WITHDRAWN	-	-
NCT00589186	Autologous DCs transduced with AD5F35-LMP-1/LMP-2	Nasopharyngeal Cancer	II	UNKNOWN	LMP-1, LMP-2	Celecoxib
NCT01149902	Autologous immature DC	HNSCC	I	COMPLETED	-	Cyclophosphamide, Docetaxel, OK-432
NCT01821495	DC-CIK Immunotherapy	Nasopharyngeal Cancer	II	UNKNOWN	–	Chemoradiotherapy
NCT03047525	DC-CIK Immunotherapy	Nasopharyngeal Carcinoma, Colorectal Cancer, Renal Cell Carcinoma, Lung Cancer	I/II	UNKNOWN	-	-
NCT03282617	Autologous CD137L-DC-EBV-VAX	Nasopharyngeal Cancer	I	COMPLETED	Epstein Barr nuclear antigen 1, LMP-1, LMP-2	–
NCT03789097	*In situ* DC vaccination with Flt3L and Poly-ICLC	HNSCC, Non-Hodgkin’s Lymphoma, Metastatic Breast Cancer	I/II	RECRUITING	Not Applicable	Radiotherapy, Pembrolizumab
NCT04166006	Autologous DC vaccination	HNSCC, neuroendocrine tumors, soft tissue sarcoma	II	RECRUITING	Autologous tumor homogenate	IL-2
NCT04476641	DC-CIK Immunotherapy	Nasopharyngeal Cancer, liver cancer, kidney cancer, lung cancer, colorectal cancer, breast cancer	II	UNKNOWN	-	-
NCT05261750	Autologous DCs and allogenic DC secretomes	Nasopharyngeal Cancer	I/II	UNKNOWN	–	Chemoradiotherapy
NCT06016920	*In situ* DC vaccination with VB10.16	HNSCC	I/II	RECRUITING	-	Pembrolizumab
NCT06097793	KSD-101	Nasopharyngeal Cancer	I	RECRUITING	EBV-related antigen	–
NCT06370026	KSD-101	Nasopharyngeal Cancer	I	NOT YET RECRUITING	EBV-related antigen	-
NCT06752473	KSD-101	Nasopharyngeal Cancer	I	NOT YET RECRUITING	EBV-related antigen	–

HNSCC, head and neck squamous carcinoma; AD5F35, adenoviral vector 5F35; LMP-1, latent membrane protein 1; LMP-2, latent membrane protein-2; CIK, cytokine-induced killer cells; CD137L-DC-EBV-VAX, autologous CD137L-DC pulsed with EBV peptides spanning Epstein Barr nuclear antigen 1, LMP1 and LMP2; ICIs, Immune Checkpoint Inhibitors; -, Not Applicable.

Chemotherapy and radiotherapy have been shown to enhance the efficacy of DC-based immunotherapy through several mechanisms. First, the abscopal effect of radiotherapy and chemotherapy can damage tumors at unirradiated sites, generating large amounts of extracellular damage-associated molecular patterns and IFN-γ. This facilitates the uptake of antigens by iDCs. Second, radiotherapy induces immunogenic cell death of immunosuppressive cell subsets within the TME, thereby blocking inhibitory molecules. Third, radiation triggers the upregulation of adhesion molecules on the vascular endothelium of tumor cells, promoting T cell infiltration (141, 142). It has been proven that the combination of radiotherapy or chemoradiation with DC injections induced strong systemic antitumor effect, and resulted in improved survival and tumor regression in SCC-tumor-bearing mice models (141, 143). Similar to chemotherapy and radiotherapy, the products released by dead tumor cells triggered by photodynamic therapy are potent stimulators for DC maturation. The combination strategy of DC vaccines and photodynamic therapy has been tested in HNSCC tumor-bearing mice models. Activation of iDCs and tumor regression were observed. Moreover, this combined therapy enhanced the anti-tumor effect of anti-PD-L1 (144).

Cholesterol metabolism is closely related to T cell function, and acyl-CoA: cholesterol acyltransferase 1 (ACAT1) inhibitors improve the anti-tumor function of CD8+ T cells by inhibiting cholesterol esterification in these cells (145). Therefore, DC vaccines can be combined with ACAT1 inhibitor therapy to simultaneously enhance CD8+ T cell-mediated anti-tumor immune responses through both mechanisms. In an animal study, a cancer stem cell antigens-loaded DC (CSCs-DC) vaccine was combined with an ACAT1 inhibitor to test the improvement of this treatment for HNSCC tumor recurrence and post-operative metastasis. Mice that received this combined treatment exhibited significantly longer survival and smaller tumor sizes (*P*< 0.01), suggesting that the ACAT1 inhibitor enhances the effect of the CSCs-DC vaccine against postoperative tumor recurrence. ACAT1 may serve as an excellent adjuvant for DC vaccines to help them better exert their anti-tumor effects (146).

### 4.3 Ongoing clinical trials of DC vaccines

There are currently 17 studies of DC-based immunotherapy for HNSCC registered on ClinicalTrials.gov (147). One of the studies is a preclinical animal experiment that involves human, and therefore is excluded from this review. Among the remaining 16 human trials (**Table 1**), 8 are in phase II. These studies cover both major DC vaccine approaches, aiming to explore the efficacy and safety of the therapies. As of today, 4 of these studies have been completed, 2 have been terminated or withdrawn, and 4 are currently recruiting participants. Additionally, a clinical trial (UMIN 000027279) registered in the University Hospital Medical Information Network (UMIN) in Japan (114) cannot be found on ClinicalTrail.gov; and a terminated pilot/feasibility approved by the Institutional Review Board (IRB) at the University of Pittsburgh and by the Food and Drug Administration (FDA) (IND #010318) (148) is also not registered on clinicaltrail.gov. Most of the studies employed the ex vivo-generated DC vaccine approach, with only 1 study (NCT06016920) utilizing the *in situ* vaccination approach. In some studies, DC therapy has been combined with radiotherapy, chemotherapy, and other immunotherapies. The completed studies to date have shown that DC therapies hold promising therapeutic potential, such as prolonged tumor-free survival, reduced relapses, improved survival rates, and enhanced biosafety. Moreover, for patients with treatment resistance, the combination of DC therapy can effectively reverse the situation.

#### 4.3.1 Flt3L/CDX-301 in combination of radiotherapy, pembrolizumab, and poly-ICLC

A phase I/II trial (NCT03789097) for HNSCC is designed to assess the safety and efficacy of a combination of four therapies: Flt3L/CDX-301, radiotherapy, Pembrolizumab, and Poly-ICLC. Radiotherapy can damage tumor cells and cause the release of tumor antigens, which can be uptake by DCs (142). Flt3L/CDX-301 is an immune cell growth factor that stimulates the production of DCs. Fms-like tyrosine kinase-3 ligand (Flt3L) induces DC proliferation, differentiation, development, and mobilization in the bone marrow, peripheral blood, and lymphoid organs by binding to the Flt3 receptor expressed on DCs (149). CDX-301 has the same amino acid sequence and comparable biologic activity as recombinant human Flt3L and specifically binds to Flt3L to promote DC activation (150). Poly-ICLC is a TLR3 agonist that can be used as a vaccine adjuvant to assist in the activation of DCs and has been shown to promote T cell infiltration into the tumor parenchyma (151). Pembrolizumab inhibits PD-1 and restores T cell functions. Together, the combination of these four therapies targets the anti-tumor response from antigen uptake to the induction of CTLs, fighting HNSCC through four distinct yet interrelated mechanisms.

#### 4.3.2 VB10.16

VB10.16 is a DNA-based DC vaccine designed for the treatment of malignant lesions associated with HPV16. It contains E6 and E7 tumor-specific antigens that are expressed by HPV16-infected cells. The mutation-inactivated E6 and E7 proteins are linked in a dimeric form to the natural human chemokine (C-C motif) ligand 3-like 1 (CCL3L1). CCL3L1 attracts DCs and binds to their receptor CCR5, thereby delivering the E6 and E7 antigens directly to DCs. This results in an increased antigenic load and enhanced cross-presentation. Mature DCs then migrate to the lymph nodes, where they activate antigen-specific T cells. This unique mechanism of targeting antigens to chemokine receptors on DCs induces a robust cellular immune response, which is superior to that of traditional therapeutic vaccines that deliver only the antigens (152). A multi-center phase I/II trial (NCT06016920) is aimed at evaluating the safety, immunogenicity, and anti-tumor activity of VB10.16 in combination with pembrolizumab in patients with unresectable recurrent or metastatic HPV16-positive HNSCC. The trial commenced on September 19, 2023, and is planned to produce primary outcomes by 2027.

#### 4.3.3 p53 peptide-loaded DC vaccines

The p53 gene is one of the most frequently mutated genes in HNSCC, with a mutant rate of 65–85% (153). Wild-type p53 (wtp53) is a key tumor suppressor protein and transcription factor that regulates cell cycle progression. It inhibits the cell cycle through multiple mechanisms, such as upregulating the expression of p21 protein, which binds to the cell cycle proteins E/Cdk2 and D/Cdk4, leading to cell cycle G1 phase blockade (154). Mutant p53 proteins often accumulate in HNSCC tumor cells, they lose their ability to regulate the cell cycle, apoptosis and DNA repair, but also frequently acquire oncogenic gain-of-functions, leading to cellular carcinogenesis (155).

A DC vaccine targeting p53 strategy is developed as an adjuvant therapy for HNSCC, and it has been tested in a phase I clinical trial (NCT00798655). In the trial, 16 patients with advanced HNSCC who were disease-free after surgery received the vaccine and were followed for up to 42 months. The 2-year and 3-year disease-free survival rates reached 88% and 80%, respectively. A decrease in Tregs was observed after vaccination, which may have been a contributing to the improved prognosis. However, the current rate of CTL-specific positive immune response to the vaccine stands at only 69%, indicating that further experiments are necessary to explore and develop strategies to enhance the positive immune response rate (115).

#### 4.3.4 WT1 peptide-loaded DC vaccines

A phase I/II pilot study (UMIN 000027279) conducted in Japan investigated the value and efficacy of DC vaccines loaded with Wilms tumor 1 (WT1) peptide in clinical settings. WT1 is frequently overexpressed in HNSCC and has an impact on cell proliferation through multiple genes involved in cell proliferation, cell cycle regulation, and DNA replication (156), and is closely associated with poor tumor differentiation and high tumor stage in HNSCC histology (157). Therefore, WT1 is a potential target for HNSCC immunotherapy. Meanwhile, OK-432 was also introduce as an adjuvant. OK-432, also called Picibanil, is a mixture of group A streptococcus with anti-tumor properties. Previous studies have shown that injection of OK-432 into HNSCC tumors resulted in tumor size reduction (158, 159). And because it is a TLR4 ligan it can serve as a adjuvant for DC vaccination (160). The study involved 11 patients with relapsed or refractory WT1+ HNSCC. They received a combination of WT1-based DC vaccine and OK-432 injection along with chemotherapy, radiotherapy, or surgery. Unfortunately, only 5 out of the 11 patients maintained a stable condition after vaccination, while the remaining 6 patients experienced varying degrees of disease progression. An increased Th1/Th2 ratio was detected after vaccination, yet, there was no significant change in other T cell subsets. In addition, CD8+T cells are found increased only in disease stable patients, suggesting that the disease progression after vaccination might be attributed to the lack of antigen-specific CTL responses (114).

The main adverse reactions observed were injection site erythema and low-grade fever. Other symptoms commonly seen with chemotherapy, such as malaise, constipation, nausea, and anorexia, were also observed. It is worth noting that two patients experienced grade 2 to 3 leukocytopenia after receiving the vaccine. However, there is still a lack of evidence to determine whether the adverse reactions were caused by chemotherapy or the DC vaccine itself (114).

#### 4.3.5 KSD-101

KSD-101 an autologous DC vaccine, loaded with EBV-associated tumor antigens derived from human monocytes. It was initially applied in the treatment of EBV-related hematological diseases, and demonstrated promising therapeutic efficacy in an ongoing early Phase I exploratory trials (NCT05635591), with efficient induction of immune responses, and both overall response rate and complete response rate of 100% (111). Administered subcutaneously, the vaccine activates EBV-specific CTLs in the body to effectively recognize and kill tumor cells. Activations of CD3+HLA-DR+T cells, CD8+T cells, CD4+T cells, and NK cells were also observed. Consequently, its application has now extended to the treatment of EBV-related NPC, and patients are being recruited for an early Phase I exploratory trial (NCT06097793) being conducted at Tongji Hospital in China.

EBV-related NPC is one of the most metastatic cancers of the head and neck, often metastasizing to lymph nodes and distant organs by the time of diagnosis. The survival rate for patients in advanced stages is only 50-60%. To date, no targeted therapies for EBV-related NPC have been approved for treatment. KSD-101 is one of the few therapies currently available for targeting EBV-related cancers and has shown more promising results. Its development is significant for the treatment of this EBV-related NPC carcinoma.

#### 4.3.6 DC-CIK vaccines

Combining DCs and cytokine-induced killer (CIK) cells for vaccination represents an innovative therapeutic strategy. CIK cells belong to a unique subpopulation of immunomodulatory cells and are classified as a type of adoptive T cell-mediated immunotherapy. They consist of CD3+/CD56+ natural killer T cells, which serve as the primary effector cells, along with CD3-/CD56+ NK cells and CD3+/CD56- cytotoxic T cells. CIK cells exhibit enhanced *in vitro* proliferation and expansion capabilities, as well as improved migratory and invasive abilities. They can induce tumor cell death through various pathways and mechanisms, demonstrating significant anti-tumor potential and a broad anti-tumor spectrum (161). Additionally, the interaction between DCs and NK cells is complex, characterized by bidirectional crosstalk. NK cells aid in DC maturation and T cell priming, while DCs can induce the maturation of NK cells and enhance their cytotoxicity. IL-15 expressed by DCs promotes NK cell proliferation, while IL-18, in conjunction with the synergistic effect of IL-12, contributes to the enhancement of NK cell cytotoxicity (162).

There are several clinical studies (NCT01821495, NCT04476641, and NCT03047525) are currently combining DC-therapy with CIK-therapy for the treatment of NPC. These studies aim to explore the initial therapeutic effects of DC-CIK therapy on tumors, the safety of the vaccine, and improvements in the quality of life of cancer patients. However, no results have been published yet.

## 5 Discussions

### 5.1 Limitations and challenges

#### 5.1.1 The low response rate to DC vaccines limits their therapeutic effect

Among the current clinical studies conducted for HNSCC, although one study reported a 100% response rate (NCT06097793) (111), the remaining studies still demonstrated less satisfactory response rates. When considering the overall efficacy of DC vaccines in other cancer therapies, only 5–15% of patients benefit from an objective immune response (163). This is primarily due to low antigen delivery efficiency and weak DC migration ability (164), which are attributed to several major factors.

First, the effects of immunosuppressive cells, such as tumor cells, Tregs, MDSCs, and immunosuppressive cytokines on DCs in the TME cannot be effectively neutralized or segregated (11–13). Consequently, directly activating DCs *in vivo* in such an environment presents significant challenges. It is important to note that the effectiveness of DCs generated ex vivo may still be compromised by the TME following their reinjection into the body.

Second, most ex vivo DC vaccine preparations favor the use of moDCs, as they are easier to obtain. However, compared to endogenous DC subsets, moDCs may lack sufficient expression of chemokine receptors, resulting in weaker migratory capacity to regional DNLs and diminished cross-presentation and T cell-stimulating capabilities within the TME.

Third, the presence of mregDCs and pDCs with semi-mature phenotypes suggests that DCs can also induce immune tolerance (44, 165), which is a potential obstacle to the success of vaccine strategies. Taken together, these factors may combine to impair the clinical efficacy of DC vaccines.

To break the current limitations, several strategies can be considered based on recent research and advancements. Instead of relying solely on moDCs, using endogenous DC subsets such as cDC1 or cDC2, which have better migratory and cross-presentation capabilities, may improve vaccine efficacy. Combining DC Vaccines with other therapies, such as ICIs therapy, which can enhance the overall immune response by overcoming immune checkpoints and boosting T cell activation. Utilizing nanoparticle delivery systems to target DC vaccines directly to lymph nodes can improve antigen delivery efficiency and reduce systemic side effects (166). In addition, it has been shown that using adjuvants or cytokines to enhance the maturation and activation of DCs before vaccination can improve their ability to migrate to lymph nodes and present antigens (167). However, current understanding of DC vaccine adjuvant dosing is still inadequate, and the immune effects of different types of vaccines paired with adjuvants cannot be accurately predicted. Future investigations are still required.

#### 5.1.2 HNSCC tumor heterogeneity and the challenge of DC vaccine target selection

HNSCC is characterized by marked tumor heterogeneity, which presents a significant challenge for the selection of effective DC vaccine targets. This heterogeneity is evident at multiple levels, including genetic, epigenetic, and phenotypic variations among tumor cells, as well as differences in the TME across patients and even within the same patient over time (168).

Epigenetic changes in HNSCC, such as DNA methylation, chromatin remodeling, histone posttranslational covalent modifications, and effects of non-coding RNA (169), can influence gene expression profiles and the presentation of TAAs (170), making it difficult to predict which antigens will be consistently expressed and immunogenic across different tumors.

In addition, HNSCC tumors can evolve over time, acquiring new mutations and phenotypes in response to treatment or other selective pressures (171, 172). This dynamic nature means that a target antigen that is effective at one point in time may become less relevant as the tumor evolves, necessitating the development of adaptive or multitargeted DC vaccines.

Addressing these challenges requires a multifaceted approach, including the development of personalized vaccines tailored to individual patient tumors, the identification of robust and consistently expressed TAAs, and strategies to overcome the immunosuppressive TME. Future research should focus on integrating advanced genomic and proteomic analyses to better understand HNSCC heterogeneity and identify optimal targets for DC-based immunotherapies.

#### 5.1.3 Technical challenges

The development and application of DC vaccines face several technical challenges. These challenges include the efficient generation and expansion of DCs both *in vivo* and ex vivo, the optimization of antigen-loading methods to ensure robust and specific T cell activation, and the need for standardized protocols for DC vaccine production and administration (16). In addition, monitoring and evaluating the performance of DC vaccines after application in HNSCC patients remains a critical area of research, as it is essential for determining the efficacy and safety of these therapies in a clinical setting. Moreover, adjuvants are also an important component of cancer vaccines, as they enhance APC function, promote epitope spreading, and boost the host’s intrinsic anti-tumor immunity (173). Due to the high production costs and significant technical challenges, DC vaccines still cannot be produced on a large scale. Addressing these technical challenges is crucial for improving the clinical applications and the outcomes of DC vaccines in HNSCC treatment.

### 5.2 Future expectations

The field of DC-based immunotherapy for HNSCC is poised to address the limitations of current ICIs therapy. DC dysfunction in the TME is a key factor underlying the poor response rates to ICIs therapy, as it impairs T cell activation and leads to immune evasion.

Restoring DC function is essential for enhancing the efficacy of ICIs therapy. DC-based immunotherapy can directly target and modulate DCs to improve antigen presentation and T cell activation. Future research should focus on developing advanced DC therapies that enhance DC maturation and migration, and on creating personalized treatment strategies to overcome tumor heterogeneity. Synergistic combination therapies with ICIs therapy will also be crucial for maximizing anti-tumor effects.

In summary, DC-based immunotherapy holds significant promise for improving the current therapeutic approaches and outcomes of HNSCC by addressing DC dysfunction and enhancing overall immune responses.
